# Novel Methyltransferase Recognition Motif Identified in *Chania multitudinisentens* RB-25^T^ gen. nov., sp. nov.

**DOI:** 10.3389/fmicb.2016.01362

**Published:** 2016-08-31

**Authors:** Robson Ee, Yan-Lue Lim, Wai-Fong Yin, Wah-Seng See-Too, Richard J. Roberts, Kok-Gan Chan

**Affiliations:** ^1^Division of Genetics and Molecular Biology, Faculty of Science, Institute of Biological Sciences, University of MalayaKuala Lumpur, Malaysia; ^2^New England BiolabsIpswich, MA, USA

**Keywords:** SMRT sequencing, methylome, REBASE, DNA methylation, prophage, DNA methyltransferase (MTase), restriction-modification (RM) system, restriction endonuclease (REase)

## Introduction

DNA methylation, defined by the addition of a methyl group to adenine or cytosine bases in DNA catalyzed by DNA methyltransferases (MTases), is one of the most studied post-replicative DNA modification mechanism in bacteria (Roberts et al., 2003b). The three forms of nucleotide methylation identified to date are: *N*6-methyladenine(^m6^A), *N*4-methylcytosine (^m4^C), and 5-methylcytosine (^m5^C) (Gromova and Khoroshaev, 2003).

Generally, MTases can be classified into two main groups: as part of a restriction modification (RM) system in which the MTase is associated with a cognate restriction endonuclease (REase) or as a solitary MTase, which as the name suggests, serves the role of an independent MTase (Murphy et al., 2013; Roberts et al., 2015). The RM systems, which are found to occur exclusively in unicellular organisms, can be further classified into four groups based on their subunit composition, recognition sequence specificity, substrate specificity, cofactor requirements, and DNA cleavage positions (Wilson and Murray, 1991; Casadesus and Low, 2006). In brief, Type I RM systems comprise three polypeptides which form a hetero-oligomeric protein complex, namely the R (restriction), M (modification), and S (specificity) subunits, that recognizes asymmetric, and bipartite recognition sequences (Murray, 2000). Type II systems, the most ubiquitous, and simplest systems, are composed of two functionally-independent R and M genes, which are responsible for restriction and methylation activity respectively. The recognition sequences of the Type II systems are most often symmetrical, but can also be asymmetric (Wilson and Murray, 1991). Type III system also consist of two subunits, named res, and mod. The mod subunit can function independently as a MTase, but only methylates one strand of the DNA. The res subunit must form a complex with the mod subunit to express its DNA restriction activity, because the recognition specificity is encoded in the mod subunit (Wilson and Murray, 1991). The recognition sequences of the known Type III systems are asymmetric and four to six bases in length. Furthermore, when known, the res enzyme requires the presence of two unmethylated recognition sites for efficient DNA cleavage (Rao et al., 2013). Lastly, the Type IV systems are restriction enzymes that recognize and cleave only methylated DNA (Vasu and Nagaraja, 2013). The sequence specificities of the Type IV systems are not well studied. MTases can be further sub-classified according to the order of their conserved amino acid motifs, which represent the DNA binding domain, the target recognition domain (TRD) and the catalytic domain (Malone et al., 1995; Jeltsch, 2002).

RM systems are often considered as the most primitive defense mechanism of prokaryotes against the invasion of extraneous DNA elements (Wilson and Murray, 1991; Bickle, 2004). However, more recent studies have offered new insights into their additional biological roles, including genomic island stabilization, species identity maintenance, generating, and enhancing genomic diversity for host fitness and adaptability as well as the regulation of gene expression (Vasu and Nagaraja, 2013).

The development of Single Molecule Real-Time (SMRT) sequencing technology which enables the simultaneous genome-wide detection of MTase activity during genome sequencing has permitted the rapid detection of novel MTase recognition motifs in various prokaryotes including *Helicobacter pylori, Salmonella enterica, Escherichia coli*, and *Campylobacter coli* (Krebes et al., 2014; Forde et al., 2015; Lee et al., 2015; Pirone-Davies et al., 2015; Zautner et al., 2015). These data are important not only for the identification of novel types of MTases, but they also lay the groundwork for the discovery of new biological roles for DNA methylation. Furthermore, these new data on previously characterized methylomes can further extend our understanding of these RM systems.

*Chania multitudinisentens* RB-25^T^ gen. nov., sp. nov. is a fully characterized newly proposed novel genus in the family of *Enterobacteriaceae*. Isolated in a soil sample collected from a former municipal landfill site, *C. multitudinisentens* RB-25^T^ was initially misidentified as a member of the *Serratia* genus (Ee et al., 2014a). Further in-depth investigation later reclassified *C. multitudinisentens* RB-25^T^ as a novel genus (Ee et al., 2016). To date, *C. multitudinisentens* RB-25^T^ is only characterized for its quorum sensing properties (the production of C4-HSL, C6-HSL, and 3-oxo-C6-HSL) and its potential chitinolytic activity (Ee et al., 2014a; Lim et al., 2015b). In this study, we report a novel methyltransferase recognition motif in *C. multitudinisentens* RB-25^T^, which will serve as the foundation for future investigation of the role of methylation in the genus *Chania*.

## Materials and methods

### Genome sequencing and assembly

Genomic DNA extraction was performed using a Masterpure™ DNA purification kit (Epicentre) on pelleted bacterial cells from an overnight culture. Briefly, the bacterial cells were first transferred into a mixture of Tissue and Cell Lysis Solution with proteinase K, and subsequently treated with RNase A prior to the addition of MPC protein precipitation reagent. Pure isopropanol was used to precipitate DNA and the precipitated DNA pellet was washed twice with 75% ethanol and resuspended in Buffer EB (Qiagen). The extracted gDNA was quantified using a Qubit fluorometer (Thermo Fisher Scientific) and was subsequently examined for its integrity using 0.8% agarose gel electrophoresis. A Nanodrop™ spectrophotometer (Thermo Fisher Scientific) was used to quantify the extracted gDNA and to determine its purity. Following the “Procedure and Checklist-20 kb Template Preparation Using BluePippin™ Size-selection system” protocol (Pacific Biosciences), a size-selected SMRTbell™ library (7 kb as size-selection cut-off length) was prepared. After annealing and polymerase binding, the library was subsequently sequenced in a PacBio RSII sequencer (Pacific Biosciences) using P5-C3 chemistry on 2 SMRT cells.

*De novo* assembly of this genome was performed as described previously in which the sequenced polymerase reads underwent three major steps within the Hierarchical Genome Assembly Process (HGAP) assembly pipeline (Pacific Biosciences), namely sub-read filtering, preassembly, assembly, and consensus polishing to generate a highly accurate polished assembly (Chin et al., 2013; Ee et al., 2014b, 2015; Lim et al., 2015a). Circularity of the assembled genome, denoted by the presence of self-overlapping ends, was determined and visualized using Contiguity (Sullivan et al., 2015) and Gepard (Krumsiek et al., 2007). The overlapping ends were finally circularized using the Minimus2 pipeline included in the AMOS software package (Treangen et al., 2011).

### Genome annotation

Genome annotation was performed using three different server-based genome annotation pipelines namely: Rapid Annotation using Subsystem Technology, RAST version 2.0 (Aziz et al., 2008); Rapid Prokaryotic Genome Annotation, PROKKA (Seemann, 2014); and NCBI Prokaryotic Genome Annotation Pipeline, PGAP. Genes of interest were subsequently curated manually by comparison between the annotation results of different pipelines followed by BLAST comparison against NCBI nucleotide (nr/nt) database and NCBI Reference Sequence, RefSeq database. Conserved domain analyses and functional protein predictions were subsequently performed using a combination of various protein model databases including NCBI-curated domains; Simple Modular Architecture Research Tool, SMART (Letunic et al., 2015); Protein Families, Pfam database (Finn et al., 2014); Clusters of Orthologous Groups of proteins, COGs database (Tatusov et al., 2003); The Institute for Genomic Research's database of protein families, TIGRFAM (Haft et al., 2003); and InterPro protein families database (Mitchell et al., 2015). PHAST analysis was also performed to identify the presence of prophages in the genome (Zhou et al., 2011).

### Base modification analysis

The complete genome sequence was uploaded into the SMRT portal and was processed into an *in silico* kinetic reference. Subsequently, SMRT analysis RS_Modification_and_Motif_Analysis.1 protocol was used to perform epigenome analysis using the imported reference sequence. A default modification quality value (QV) score of 30 (correspond to a *p*-value of 0.001) was used to call the modified bases.

### RM system genes annotation

By using the SEQWARE computer resources, which constitutes BLAST-based software modules in combination with REBASE curated internal databases, the genome sequence was scanned for homologs of RM system genes (Murray et al., 2012). As described previously, the prediction were supported by sequence similarity, presence, and order of predictive functional motifs, and the known genomic context and characteristics of previously characterized RM system genes (Murray et al., 2012; Pirone-Davies et al., 2015). The predicted RM system genes were named following the proposed nomenclature as described by Roberts et al. (2003a). Assignment of the predicted RM system genes to the identified recognition motifs was performed based on putative methyltransferases sequence homology and RM system Type pairing.

## Results

### Genome characteristics

The first draft genome sequence of *C. multitudinisentens* RB-25^T^ which was deposited in GenBank with the accession number CP007044.1 was a non-contiguous single contig assembly (35-fold coverage) constructed from sequence data generated using a single SMRT cell. Here we report the updated version of the genome sequence (CP007044.2) which with the sequence data generated from an additional SMRT cell, enabled successful assembly into a single circular chromosome of 5,488,183 bp (85.4-fold coverage) with 50.90% GC content. A total of 4883 genes, 4540 coding DNA sequences, 243 pseudo genes, 3 CRISPR arrays, 22 rRNAs, 77 tRNAs, and 1 ncRNA were annotated in the genome. Genome sequence data are available at GenBank in the format of FASTA, annotated GenBank flat file, graphical, and ASN.1 formats.

Following annotation using a prophage search tool (PHAST), two intact prophages, an incomplete prophage and a questionable prophage were identified from the genome (Supplementary Tables 1–5).

### Base modification analysis

Genome-wide base modification analysis revealed a distinct population of adenine bases with high modification QV (modQV) value, as demonstrated by the distinct separation of the red adenine cloud from the background in the modQV vs coverage scatterplot (Supplementary Figure 1). Motif analysis detected a total of two methyltransferase recognition motifs. The first motif is 5′-G^m6^ATC-3′, a common motif pattern corresponding to the well-known specificity pattern for many Type II alpha subtype MTases (Barras and Marinus, 1989; Henaut et al., 1996; Low et al., 2001). Methylation of the adenine residues in this motif is known to influence the regulation of various cellular events for instance gene expression, virulence, DNA replication coordination, as well as normal cellular processes (Low et al., 2001; Murphy et al., 2013). The second motif detected is a novel recognition motif, 5′-GC^m6^AGNNNNNTCC-3′ with its partner motif 5′-GG^m6^ANNNNNCTGC-3′. This motif matched the asymmetric, and bipartite structure of a typical Type I recognition sequence with a 3 nucleotides and a 4 nucleotides component separated by a nonspecific spacer sequence (represented by N). Across the genome, more than 99% of all genomic positions, which correspond to both methyltransferase recognition motifs were found to be methylated (Supplementary Table 6).

### Restriction modification system analysis

Annotation of the *C. multitudinisentens* RB-25^T^ genome identified seven putative DNA methyltransferase-encoding genes that might be associated with Type II RM systems and one MTase gene that would be part of a Type I RM system. Among the eight annotated MTases, three were assigned to the MTase recognition motifs detected in base modification analysis, whereas another three MTases were predicted to be potentially inactive and two of the MTases had unknown specificity. Each of the identified MTases and the predicted specificities are summarized in Table 1.

**Table 1:** **MTases and their predicted specificities identified in *Chania multitudinisentens* RB-25^T^**.

**RM system genes annotated**	**Type (subtype)**	**Locus tag (bp start/stop)^a^**	**Predicted recognition motif^b^**	**Methylation type**
M.CmuRB25I	I (gamma)	Z042_16340 (5441947/5444100)	GG^m6^**A**GNNNNN**T**CC	6 mA
S.CmuRB25I	I	Z042_16335 (5444100/5445641)	GCAGNNNNNTCC	
M.CmuRB25DamP	II (alpha)	Z042_10170 (1345643/1346455)	G^m6^**AT**C	6 mA
M.CmuRB25ORF1485P	II (alpha)	Z042_01485 (3271527/3272399)	G^m6^**AT**C	6 mA
M.CmuRB25ORF14775P	II	(319513/320601)	GTCGAC	5 mC
CmuRB25ORF14775P	II (P)	(318782/319516)	GTCGAC	
M.CmuRB25ORF23095P	II	Z042_23095 (4027210/4029192)	–	–
M.CmuRB25ORF23090P	II (beta)	Z042_23090 (4029189/4030583)	AAGCTT	–
M.CmuRB25ORF23015P	II (gamma)	Z042_23015 (4038239/4038991)	–	–
M.CmuRB25ORF22915P	II (alpha)	(4049231/4050046)	TGGCCA	–
CmuRB25McrBCP	IV	Z042_16355 (5438103/5440178)	–	–

a *For predicted MTases which do not have a GenBank locus tag, genes coordinates are provided in the parenthesis (bp start/stop)*.

b *Modified bases are highlighted in bold*.

Firstly, based on the matching RM system types, the CmuRB25ORF16340P system, the only Type I RM system annotated in the genome was assigned to be responsible for the novel Type I motif, 5′-GC^m6^AGNNNNNTCC-3′. The S subunit responsible for recognizing this sequence was renamed S.CmuRB25I and its partner MTase was renamed M.CmuRB25I (Supplementary Table 7). S.CmuRB25I was a typical Type I S subunit and its N-terminal target recognition domain (TRD1) showed a reasonable similarity to the C-terminal TRD (TRD2) of S.Eco3609I (TGHAYNNNNCTNC). M.CmuRB25I was a typical *N*^6^ DNA methylase of the gamma subtype.

Secondly, two Type II solitary methyltransferases, M.CmuRB25DamP, and M.CmuRB25ORF1485P, were predicted to be the candidate enzymes responsible for formation of the G^m6^ATC motif due to their sequence similarity to various MTases which were previously predicted or documented to recognize G^m6^ATC (Supplementary Tables 8–9). Both MTases contained the characteristic D12 class *N*^6^-adenine-specific DNA methyltransferase pfam domain (pfam02086) and were grouped into COG0338 (site specific DNA-adenine methylase). M.CmuRB25DamP is potentially the constitutive G^m6^ATC methyltransferase of *C. multitudinisentens* as it showed higher sequence similarity (93%) to G^m6^ATC methyltransferases of *Serratia* species, including one of the REBASE gold standard MTases, M.SmaII in *Serratia marcescens* strain Sb. On the other hand, M.CmuRB25ORF1485P shows only 54% of amino acid identity to various G^m6^ATC methyltransferases from *Dickeya* and *Yersinia* species and was identified to be in close proximity to a predicted intact prophage sequence which led us to speculate that this gene could be a prophage-encoded MTase. Even more interesting, the MTases which have sequence similarity to M.CmuRB25ORF1485P are also MTases which were found to be located within intact prophage sequences, hence further supporting our hypothesis (data not shown). Prophage-derived Dam homologs are prevalent, examples of prophages found to encode Dam homologs include phage T4, phage P1, Shiga toxin-encoding 933 W phage, and *Pseudomonas* phage B3 (Sternberg and Coulby, 1990; Miller et al., 2003; Braid et al., 2004; Murphy et al., 2008). These Dam homologs were found to serve various function in the phage genomes, including phage genome protection role from host REases, phage packaging, and progeny release, maintenance of phage lysogeny, and regulation of phage gene expression (Sternberg et al., 1986; Sternberg and Coulby, 1990; Lobocka et al., 2004; Murphy et al., 2013). Although the majority of MTases encoded by prophages were observed to be active only during the lytic stage (Trautner et al., 1980; Citron et al., 1989), we cannot rule out the possibility that M.CmuRB25ORF1485P could also contribute to formation of G^m6^ATC methylation. Therefore, due to the presence of two possible MTase candidates for motif G^m6^ATC, no confirmed assignment was made.

Another three MTases were also predicted to recognize specific target sites based on their sequence similarity to characterized MTases. The first is M.CmuRB25ORF14775P, which is predicted to encode a ^m5^C DNA MTase that recognizes 5′-GTCGAC-3′ based on matching conserved amino acid motif pattern and 44% amino acid sequence identity to M.BbrUII, which is known to recognize GTCGAC (O'Connell Motherway et al., 2009). Other methylases of similar specificity can be found in Supplementary Table 10. Adjacent to this MTase, in a tandem transcriptional orientation with a 3 bp overlap, is predicted to be its cognate REase, CmuRB25ORF14775P. The second example is M.CmuRB25ORF23090P, a Type II, subtype beta solitary MTase which shares sequence similarity (60%) to MTases that recognize 5′-AAGCTT-3′ (Supplementary Table 11). The third one, M.CmuRB25ORF22915P, is a Type II orphan methyltransferase (subtype alpha), and is predicted to recognize 5′-TGGCCA-C′ due to its high sequence similarity (75%) to a large number of MTases (>30) predicted to recognize this target motif (Supplementary Table 12).

Lastly, two MTases, M.CmuRB25ORF23095P and M.CmuRB25ORF23015P, were annotated, but with unknown recognition sequences. Both MTases are Type II solitary MTases, in which, based on their conserved amino acid motif arrangement, M.CmuRB25ORF23095P is predicted to be a ^m5^C MTase whereas M.CmuRB25ORF23015P is an amino MTase of the gamma subtype.

### Data access

The methylome data of *C. multitudinisentens* strain RB-25^T^ are accessible through organism number 12049 in REBASE, in which the full profile of annotated RM system genes in the form of a summary table can be accessed. Through the complete genome hyperlink, various information which could aid in understanding the complete methylome of *C. multitudinisentens* is available, including: visualization of the annotated RM system genes location on a circular genome map (Supplementary Figure 2), schematics of the conserved amino acid motifs on each annotated RM system genes as well as the genomic arrangement of the associated specificity subunits or REases, summary of the detailed annotation information of each RM system genes, and the annotation report. The detailed characteristics and sequence data of all of the RM system genes detected in the genome can be viewed through the hyperlink of each gene listed on the summary table. Cross reference information through other database such as Expasy, InterPro, Pfam, ProDom, ProtoMap, and PRESAGE can also be accessed through the “sequence data” hyperlink. Details of prophages are available in the PHAST database with the NC number of CP007044.2 (Web link: http://phast.wishartlab.com/cgi-bin/Results.cgi?num=CP007044.2&multi=1).

## Author contributions

All authors listed, have made substantial, direct and intellectual contribution to the work, and approved it for publication.

### Conflict of interest statement

The authors declare that the research was conducted in the absence of any commercial or financial relationships that could be construed as a potential conflict of interest. RJR is a full-time employee of New England Biolabs, a company that sells research reagents such as DNA MTases.
