# Supplementary material for: Novel Methyltransferase Recognition Motif Identified in Chania multitudinisentens RB-25T gen. nov., sp. nov
Source: Front Microbiol. 2016 Aug 31;7:1362. doi: 10.3389/fmicb.2016.01362 (PMC5005818; doi:10.3389/fmicb.2016.01362)
Supplement: Supplementary file 1 [file DataSheet1.DOC]

**Supplementary Figure 1.** Modification QV vs coverage scatterplot which shows a distinct divergence of adenine bases (with higher modification QV) from the other three bases at a similar coverage.


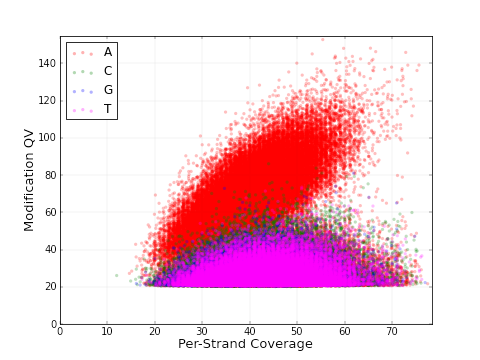


**Supplementary Figure 2.** Circular genome map of R-M system genes location in *Chania* *multitudinisentens* RB-25^T^


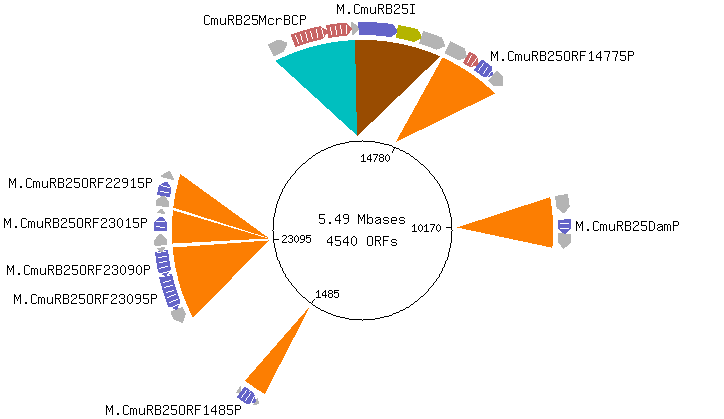


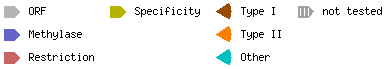


| **Region** | **Region_length** | **Completeness** | **Score** | **#cds** | **Region_position** | **Possible phage** | **Gc_percentage** |
| --- | --- | --- | --- | --- | --- | --- | --- |
| 1 | 15.7Kb | intact | 150 | 18 | 3249994-3265695 | PHAGE_Salmon_SEN5_NC_028701, | 53.20% |
| 2 | 51.8Kb | intact | 140 | 44 | 3981376-4033190 | PHAGE_Entero_VT2phi_272_NC_028656 | 49.09% |
| 3 | 30.4Kb | incomplete | 30 | 22 | 4023102-4053533 | PHAGE_Vibrio_12A4_NC_021068 | 50.15% |
| 4 | 21.1Kb | questionable | 70 | 25 | 5101450-5122639 | PHAGE_Salmon_SEN34_NC_028699 | 49.61% |

**Supplementary Table 1.** 4 prophage regions have been identified, of which 2 regions are intact, 1 region is incomplete, 1 region is questionable.

**Supplementary Table 2. Intact prophage in region 1, total: 18 CDS.**

| **#** | **CDS_POSITION** | **BLAST_HIT** | **E-VALUE** |
| --- | --- | --- | --- |
| 1 | 3249994..3250422 | PROPHAGE_Salmon_Ty2: putative phage tail protein; Z042_01605; phage(gi29143763) | 6e-45 |
| 2 | complement(3250506..3251672) | PHAGE_Salmon_SEN5_NC_028701: tail fiber protein; Z042_26010; phage(gi966201574) | 5e-51 |
| 3 | complement(3251681..3254680) | PHAGE_Burkho_phiE255_NC_009237: gp26; Z042_01595; phage(gi134288791) | 2e-07 |
| 4 | complement(3254682..3255281) | PHAGE_Entero_Fels_2_NC_010463: P2 gpI-like baseplate assembly protein; Z042_01590; phage(gi169936031) | 7e-56 |
| 5 | complement(3255274..3256185) | PHAGE_Erwini_ENT90_NC_019932: baseplate assembly protein; Z042_01585; phage(gi431810943) | 5e-111 |
| 6 | complement(3256182..3256532) | PHAGE_Escher_pro147_NC_028896: baseplate assembly protein W; Z042_01580; phage(gi971753732) | 2e-26 |
| 7 | complement(3256529..3257083) | PHAGE_Salmon_SEN5_NC_028701: baseplate assembly protein V; Z042_01575; phage(gi966201570) | 2e-35 |
| 8 | complement(3257180..3257809) | PHAGE_Salmon_SEN5_NC_028701: tail completion protein; Z042_01570; phage(gi966201568) | 1e-39 |
| 9 | complement(3257806..3258297) | PHAGE_Salmon_SEN5_NC_028701: tail protein; Z042_01565; phage(gi966201567) | 3e-38 |
| 10 | complement(3258906..3259481) | PHAGE_Pseudo_vB_PaeM_PS24_NC_028882: hypothetical protein; Z042_01555; phage(gi971752090) | 1e-51 |
| 11 | complement(3259459..3259770) | PHAGE_Entero_epsilon15_NC_004775: holin; Z042_26020; phage(gi30387403) | 6e-13 |
| 12 | complement(3259760..3260146) | PHAGE_Burkho_KS5_NC_015265: gp35; Z042_01545; phage(gi327198033) | 8e-12 |
| 13 | complement(3260181..3260387) | PHAGE_Entero_fiAA91_ss_NC_022750: tail protein; Z042_01540; phage(gi557307592) | 4e-16 |
| 14 | complement(3260387..3260881) | PHAGE_Salmon_SEN5_NC_028701: head completion-stabilization protein; Z042_01535; phage(gi966201562) | 3e-36 |
| 15 | complement(3260979..3261830) | PHAGE_Salmon_SEN5_NC_028701: terminase endonuclease subunit; Z042_01530; phage(gi966201561) | 4e-53 |
| 16 | complement(3261886..3262920) | PHAGE_Salmon_SEN5_NC_028701: major capsid protein; Z042_01525; phage(gi966201560) | 2e-123 |
| 17 | complement(3262957..3263817) | PHAGE_Salmon_SEN5_NC_028701: capsid scaffolding protein; Z042_01520; phage(gi966201559) | 1e-70 |
| 18 | 3263974..3265695 | PHAGE_Salmon_SEN5_NC_028701: terminase ATPase subunit; Z042_01515; phage(gi966201558) | 0.0 |

**Supplementary Table 3. Intact prophage in region 2, total: 44 CDS.**

| **#** | **CDS_POSITION** | **BLAST_HIT** | **E-VALUE** |
| --- | --- | --- | --- |
| 1 | 3981376..3981387 | attL    GGAGGTGGCTGA | 0.0 |
| 2 | 3983248..3983260 | attL    ATACAAAATAAGT | 0.0 |
| 3 | complement(3987440..3988246) | PROPHAGE_Xantho_33913: ISxcC1 transposase; Z042_23325; phage(gi21231060) | 5e-81 |
| 4 | complement(3988282..3988548) | PROPHAGE_Salmon_LT2: transposase; Z042_23320; phage(gi16766077) | 7e-32 |
| 5 | 3988763..3989221 | cytoplasmic protein; Z042_23315 | 0.0 |
| 6 | 3989206..3989538 | cytoplasmic protein; Z042_23310 | 0.0 |
| 7 | 3989607..3990131 | PROPHAGE_Ralsto_GMI1000: ISRSO11-transposase ORFA protein; Z042_23305; phage(gi17546156) | 3e-23 |
| 8 | 3990125..3990955 | PROPHAGE_Ralsto_GMI1000: isrso11-transposase orfb protein; Z042_23300; phage(gi17546155) | 3e-87 |
| 9 | complement(3991914..3992279) | PHAGE_Cronob_phiES15_NC_018454: putative tail tape measure protein; Z042_23290; phage(gi401817605) | 9e-11 |
| 10 | complement(3992400..3992630) | hypothetical protein; Z042_23285 | 0.0 |
| 11 | 3992841..3992853 | attR    ATACAAAATAAGT | 0.0 |
| 12 | complement(3992866..3993078) | PHAGE_Cronob_phiES15_NC_018454: hypothetical protein; Z042_23280; phage(gi401817604) | 2e-21 |
| 13 | 3993327..3993689 | PHAGE_Entero_mEp235_NC_019708: integrase; Z042_23270; phage(gi428781836) | 8e-23 |
| 14 | 3993937..3994149 | hypothetical protein; Z042_23265 | 0.0 |
| 15 | 3994220..3994450 | hypothetical protein; Z042_23260 | 0.0 |
| 16 | complement(3994458..4001903) | PHAGE_Escher_P13374_NC_018846: hypothetical protein; Z042_26275; phage(gi410491673) | 0.0 |
| 17 | 4002671..4002934 | hypothetical protein; Z042_23245 | 0.0 |
| 18 | complement(4003020..4004351) | PHAGE_Entero_VT2phi_272_NC_028656: hypothetical protein; Z042_23240; phage(gi966197795) | 1e-27 |
| 19 | complement(4004361..4004621) | PHAGE_Entero_VT2phi_272_NC_028656: hypothetical protein; Z042_23235; phage(gi966197794) | 6e-16 |
| 20 | complement(4004621..4005007) | PHAGE_Entero_VT2phi_272_NC_028656: hypothetical protein; Z042_23230; phage(gi966197793) | 2e-32 |
| 21 | complement(4005016..4005696) | PHAGE_Entero_VT2phi_272_NC_028656: hypothetical protein; Z042_23225; phage(gi966197792) | 2e-52 |
| 22 | complement(4005704..4006075) | PHAGE_Shigel_Ss_VASD_NC_028685: hypothetical protein; Z042_23220; phage(gi966200490) | 4e-28 |
| 23 | complement(4007343..4008968) | PHAGE_Shigel_POCJ13_NC_025434: hypothetical protein; Z042_23205; phage(gi725949760) | 3e-164 |
| 24 | complement(4008976..4012305) | PHAGE_Entero_VT2phi_272_NC_028656: putative tail fiber protein; Z042_23200; phage(gi966197781) | 9e-12 |
| 25 | complement(4012302..4012961) | PHAGE_Entero_VT2phi_272_NC_028656: hypothetical protein; Z042_23195; phage(gi966197780) | 5e-54 |
| 26 | complement(4012962..4013558) | PHAGE_Entero_VT2phi_272_NC_028656: hypothetical protein; Z042_23190; phage(gi966197779) | 4e-30 |
| 27 | complement(4013558..4014040) | PHAGE_Entero_VT2phi_272_NC_028656: hypothetical protein; Z042_23185; phage(gi966197778) | 1e-31 |
| 28 | complement(4014103..4014486) | PHAGE_Entero_VT2phi_272_NC_028656: hypothetical protein; Z042_23180; phage(gi966197777) | 2e-20 |
| 29 | complement(4014546..4015769) | PHAGE_Entero_VT2phi_272_NC_028656: hypothetical protein; Z042_23175; phage(gi966197776) | 5e-177 |
| 30 | complement(4015834..4016901) | PHAGE_Entero_VT2phi_272_NC_028656: hypothetical protein; Z042_23170; phage(gi966197775) | 7e-63 |
| 31 | complement(4017171..4019294) | PHAGE_Entero_VT2phi_272_NC_028656: putative portal protein; Z042_23160; phage(gi966197774) | 0.0 |
| 32 | complement(4019294..4021009) | PHAGE_Entero_VT2phi_272_NC_028656: large subunit terminase; Z042_23155; phage(gi966197773) | 0.0 |
| 33 | complement(4021006..4021869) | PHAGE_Entero_VT2phi_272_NC_028656: terminase small subunit; Z042_23150; phage(gi966197772) | 6e-48 |
| 34 | complement(4021943..4022149) | PHAGE_Citrob_Moon_NC_027331: hypothetical protein; Z042_26280; phage(gi849248798) | 9e-08 |
| 35 | complement(4022497..4023039) | PHAGE_Pectob_ZF40_NC_019522: hypothetical protein; Z042_23140; phage(gi422936675) | 3e-22 |
| 36 | complement(4023172..4023714) | PHAGE_Vibrio_8_NC_022747: putative endolysin; Z042_23135; phage(gi557307506) | 2e-27 |
| 37 | complement(4023711..4023944) | hypothetical protein; Z042_23130 | 0.0 |
| 38 | complement(4024091..4024483) | hypothetical protein; Z042_23125 | 0.0 |
| 39 | complement(4024526..4025341) | hypothetical protein; Z042_23120 | 0.0 |
| 40 | complement(4025345..4025734) | PHAGE_Psychr_pOW20_A_NC_020841: hypothetical protein; Z042_23115; phage(gi472339811) | 7e-11 |
| 41 | complement(4025781..4026113) | PHAGE_Vibrio_VvAW1_NC_020488: protein of unknown function (DUF1364); Z042_23110; phage(gi460042926) | 1e-17 |
| 42 | complement(4026106..4026531) | PHAGE_Stx2_converting_I_NC_003525: hypothetical protein Stx2Ip073; Z042_23105; phage(gi20065868) | 4e-07 |
| 43 | complement(4026542..4027213) | PHAGE_Entero_mEp237_NC_019704: hypothetical protein; Z042_23100; phage(gi435439315) | 2e-21 |
| 44 | complement(4027210..4029192) | PHAGE_Pectob_ZF40_NC_019522: putative methylase; Z042_23095; phage(gi422936661) | 0.0 |
| 45 | complement(4029189..4030583) | PHAGE_Salmon_9NA_NC_025443: putative DNA methylase; Z042_23090; phage(gi712914017) | 2e-92 |
| 46 | complement(4030580..4030825) | hypothetical protein; Z042_23085 | 0.0 |
| 47 | complement(4030822..4031961) | PHAGE_Entero_P1_NC_005856: HrdC; Z042_23080; phage(gi46401690) | 1e-82 |
| 48 | 4033190..4033201 | attR    GGAGGTGGCTGA | 0.0 |

**Supplementary Table 4. Incomplete prophage in region 3, total: 22 CDS.**

| **#** | **CDS_POSITION** | **BLAST_HIT** | **E-VALUE** |
| --- | --- | --- | --- |
| 1 | 4023102..4023114 | attL    ATTACAGATTTCA | 0.0 |
| 2 | 4037454..4038053 | PHAGE_Shigel_POCJ13_NC_025434: hypothetical protein; Z042_26310; phage(gi725949711) | 4e-40 |
| 3 | 4038239..4038991 | PHAGE_Thalas_BA3_NC_009990: hypothetical protein BA3_0002; Z042_23015; phage(gi160700596) | 7e-29 |
| 4 | 4039100..4039378 | hypothetical protein; Z042_23010 | 0.0 |
| 5 | 4039662..4039925 | PHAGE_Cellul_phi18:3_NC_021794: KTSC domain containing protein; Z042_23005; phage(gi526177431) | 6e-05 |
| 6 | 4039990..4040322 | hypothetical protein; Z042_23000 | 0.0 |
| 7 | 4040622..4041455 | PHAGE_Ralsto_RSK1_NC_022915: putative phage recombination protein; Z042_26315; phage(gi560186064) | 4e-69 |
| 8 | 4041442..4043028 | PHAGE_Thalas_BA3_NC_009990: hypothetical protein BA3_0032; Z042_22985; phage(gi160700626) | 1e-67 |
| 9 | 4043069..4044223 | PHAGE_Vibrio_12A4_NC_021068: hypothetical protein; Z042_22980; phage(gi481019127) | 3e-43 |
| 10 | 4044225..4044647 | hypothetical protein; Z042_22975 | 0.0 |
| 11 | 4045286..4045480 | hypothetical protein; Z042_26325 | 0.0 |
| 12 | 4045490..4045762 | PHAGE_Entero_HK225_NC_019717: hypothetical protein; Z042_22965; phage(gi428782414) | 1e-08 |
| 13 | 4045752..4045985 | PHAGE_Erwini_PEp14_NC_016767: hypothetical protein; Z042_22960; phage(gi374531888) | 3e-21 |
| 14 | 4046488..4047012 | PHAGE_Entero_Mu_NC_000929: hypothetical protein Mup12; Z042_22950; phage(gi9633502) | 5e-14 |
| 15 | 4047009..4047221 | hypothetical protein; Z042_22945 | 0.0 |
| 16 | 4047236..4047580 | PHAGE_Vibrio_VvAW1_NC_020488: hypothetical protein; Z042_22940; phage(gi460042924) | 6e-21 |
| 17 | 4047965..4048657 | PHAGE_Serrat_Eta_NC_021563: hypothetical protein; Z042_22925; phage(gi514361067) | 2e-43 |
| 18 | 4048654..4049223 | prophage PSSB64-02; Z042_26330 | 0.0 |
| 19 | 4049216..4050046 | PHAGE_Pseudo_F116_NC_006552: DNA adenine methyltransferase; Z042_22915; phage(gi56692911) | 8e-71 |
| 20 | 4050056..4050574 | PHAGE_Yersin_PY54_NC_005069: hypothetical protein PY54p47; Z042_22910; phage(gi33770556) | 4e-49 |
| 21 | 4051546..4051815 | PHAGE_Entero_mEp235_NC_019708: excisionase; Z042_22900; phage(gi428781837) | 8e-07 |
| 22 | 4051790..4052875 | PHAGE_Entero_mEp235_NC_019708: integrase; Z042_22895; phage(gi428781836) | 2e-75 |
| 23 | complement(4053192..4053533) | PHAGE_Salmon_SEN1_NC_029003: hypothetical protein; Z042_22890; phage(gi971764937) | 1e-09 |
| 24 | 4053520..4053532 | attR    ATTACAGATTTCA | 0.0 |

**Supplementary Table 5. Questionable prophage in region 4, total: 25 CDS.**

| **#** | **CDS_POSITION** | **BLAST_HIT** | **E-VALUE** |
| --- | --- | --- | --- |
| 1 | complement(5101450..5102019) | PHAGE_Pseudo_vB_PaeM_PAO1_Ab03_NC_026587: putative peptidoglycan hydrolase, endolysin; Z042_17910; phage(gi764159774) | 2e-53 |
| 2 | complement(5102012..5102317) | PHAGE_Escher_Pollock_NC_027381: holin/antiholin; Z042_17905; phage(gi849254839) | 6e-13 |
| 3 | complement(5102307..5102687) | PHAGE_Burkho_KS5_NC_015265: gp35; Z042_17900; phage(gi327198033) | 9e-16 |
| 4 | complement(5104554..5105609) | PHAGE_Entero_JenK1_NC_029021: tail fiber protein; Z042_17890; phage(gi971766395) | 6e-22 |
| 5 | complement(5105777..5106655) | PHAGE_Klebsi_JD001_NC_020204: putative tail-fiber protein; Z042_17885; phage(gi448245166) | 2e-27 |
| 6 | complement(5106672..5107262) | PHAGE_Escher_pro483_NC_028943: putative tail fiber assembly protein; Z042_17880; phage(gi971758533) | 2e-42 |
| 7 | complement(5107262..5107975) | PHAGE_Entero_HK106_NC_019768: side tail fiber protein; Z042_17875; phage(gi428783303) | 1e-38 |
| 8 | complement(5107968..5108636) | PHAGE_Salmon_SEN34_NC_028699: hypothetical protein; Z042_17870; phage(gi966201443) | 7e-47 |
| 9 | complement(5108633..5109817) | PHAGE_Salmon_SEN34_NC_028699: hypothetical protein; Z042_17865; phage(gi966201442) | 6e-124 |
| 10 | complement(5109814..5110167) | PHAGE_Salmon_SEN34_NC_028699: hypothetical protein; Z042_17860; phage(gi966201441) | 1e-36 |
| 11 | complement(5110167..5110922) | PHAGE_Salmon_SEN34_NC_028699: baseplate assembly protein; Z042_17855; phage(gi966201439) | 6e-84 |
| 12 | complement(5110912..5111862) | PHAGE_Salmon_SEN34_NC_028699: hypothetical protein; Z042_17850; phage(gi966201436) | 1e-70 |
| 13 | complement(5111901..5112200) | PHAGE_Salmon_SEN34_NC_028699: hypothetical protein; Z042_17845; phage(gi966201435) | 2e-17 |
| 14 | complement(5112197..5112832) | PHAGE_Salmon_SEN34_NC_028699: hypothetical protein; Z042_17840; phage(gi966201434) | 2e-47 |
| 15 | complement(5112832..5114430) | hypothetical protein; Z042_17835 | 0.0 |
| 16 | complement(5114693..5115091) | PHAGE_Salmon_SEN34_NC_028699: hypothetical protein; Z042_17830; phage(gi966201432) | 2e-31 |
| 17 | complement(5115094..5115534) | PHAGE_Salmon_SEN34_NC_028699: hypothetical protein; Z042_17825; phage(gi966201431) | 1e-51 |
| 18 | complement(5115550..5117022) | PHAGE_Salmon_SEN34_NC_028699: hypothetical protein; Z042_17820; phage(gi966201430) | 1e-84 |
| 19 | complement(5117027..5117542) | PHAGE_Salmon_SEN34_NC_028699: hypothetical protein; Z042_17815; phage(gi966201429) | 2e-35 |
| 20 | complement(5117598..5117999) | PHAGE_Salmon_SEN34_NC_028699: hypothetical protein; Z042_17810; phage(gi966201428) | 4e-43 |
| 21 | complement(5118072..5118482) | PHAGE_Salmon_SEN34_NC_028699: hypothetical protein; Z042_17805; phage(gi966201426) | 1e-41 |
| 22 | complement(5118485..5118709) | hypothetical protein; Z042_17800 | 0.0 |
| 23 | complement(5120506..5120865) | PHAGE_Shigel_SfIV_NC_022749: antitermination Q; Z042_17790; phage(gi557307572) | 4e-12 |
| 24 | 5121007..5121717 | PHAGE_Pseudo_vB_PaeP_Tr60_Ab31_NC_023575: Putative Cro/CI transcriptional regulator; Z042_17785; phage(gi589286922) | 4e-34 |
| 25 | complement(5121905..5122639) | PHAGE_Bacill_TsarBomba_NC_028890: putative phosphoadenosine phosphosulfate reductase; Z042_17780; phage(gi971752994) | 2e-05 |

**Supplementary Table 6.** Summary of the detected methylated position of each MTase recognition motif across the genome.

| **Motifs** | **Type** | **% Motifs detected** | **Number of motifs detected** | **Number of motifs in genome** | **Mean modification QV** | **Mean Motif coverage** | **Partner Motif** |
| --- | --- | --- | --- | --- | --- | --- | --- |
| 5’-GC**A**GNNNNNTCC-3’ | m6A | 99.86% | 694 | 695 | 68.36 | 39.50 | 5’-GG**A**NNNNNCTGC-3’ |
| 5’-GG**A**NNNNNCTGC-3’ | m6A | 99.57% | 692 | 695 | 67.77 | 39.84 | 5’-GC**A**GNNNNNTCC-3’ |
| 5’-G**A**TC-3’ | m6A | 99.74% | 54734 | 54878 | 71.84 | 39.90 | 5’-G**A**TC-3’ |

**Supplementary Table 7.** Top BLASTX hits of M.CmuRB25ORF16340P against the REBASE database

| **MTase** | **Length** | **Type** | **Specificity** | **Score** | **E-value** | **Identity** |
| --- | --- | --- | --- | --- | --- | --- |
| M.PanB19ORFGP | 717 | 1 | - | 705 | 0.00E+00 | 84% |
| M.Eco3317ORF6190P | 715 | 1 | - | 701 | 0.00E+00 | 84% |
| M.EcoWORF4661P | 715 | 1 | - | 700 | 0.00E+00 | 84% |
| M.EcoLY180ORF22570P | 715 | 1 | - | 700 | 0.00E+00 | 84% |
| M.EcoKO11ORF4012P | 715 | 1 | - | 700 | 0.00E+00 | 84% |
| M.EcoFlORF23145P | 715 | 1 | - | 700 | 0.00E+00 | 84% |
| M.Eco8073ORFBP | 715 | 1 | - | 700 | 0.00E+00 | 84% |
| M.Eco26777ORF19250P | 715 | 1 | - | 700 | 0.00E+00 | 84% |
| M.Eco10182ORF2865P | 715 | 1 | - | 700 | 0.00E+00 | 84% |
| M.EcoSO63ORF26875P | 715 | 1 | TGHAYNNNNCTNC | 699 | 0.00E+00 | 84% |
| M.EcoSO17ORF9200P | 715 | 1 | TGHAYNNNNCTNC | 699 | 0.00E+00 | 84% |
| M.EcoSO157ORF17925P | 715 | 1 | TGHAYNNNNCTNC | 699 | 0.00E+00 | 84% |
| M.EcoS6400ORFAP | 715 | 1 | - | 699 | 0.00E+00 | 84% |
| M.EcoCE10ORF5048P | 715 | 2 | - | 699 | 0.00E+00 | 84% |
| [M.Eco3609I](http://rebase.neb.com/cgi-bin/seqget?M.Eco3609I) | 715 | 1 | TGHAYNNNNCTNC | 699 | 0.00E+00 | 84% |
| [M.Kpn2242ORF1575P](http://rebase.neb.com/cgi-bin/seqget?M.Kpn2242ORF1575P) | 717 | 1 | - | 698 | 0.00E+00 | 84% |
| [M.Kpn1158ORF4524P](http://rebase.neb.com/cgi-bin/seqget?M.Kpn1158ORF4524P) | 717 | 1 | - | 698 | 0.00E+00 | 84% |
| [M.EfeORF3102P](http://rebase.neb.com/cgi-bin/seqget?M.EfeORF3102P) | 715 | 2 | - | 697 | 0.00E+00 | 84% |
| [M.Eco26776ORF9825P](http://rebase.neb.com/cgi-bin/seqget?M.Eco26776ORF9825P) | 715 | 1 | - | 696 | 0.00E+00 | 84% |
| [M.AspP2S70ORF2145P](http://rebase.neb.com/cgi-bin/seqget?M.AspP2S70ORF2145P) | 696 | 1 | - | 694 | 0.00E+00 | 79% |
| [M.KpnEcl8ORFAP](http://rebase.neb.com/cgi-bin/seqget?M.KpnEcl8ORFAP) | 717 | 1 | - | 687 | 0.00E+00 | 83% |
| [M.PbaC6819ORF8525P](http://rebase.neb.com/cgi-bin/seqget?M.PbaC6819ORF8525P) | 717 | 1 | - | 687 | 0.00E+00 | 81% |
| [M.PbaC6918ORF2475P](http://rebase.neb.com/cgi-bin/seqget?M.PbaC6918ORF2475P) | 717 | 1 | - | 686 | 0.00E+00 | 81% |
| [M.Vha603ORFCP](http://rebase.neb.com/cgi-bin/seqget?M.Vha603ORFCP) | 714 | 1 | - | 676 | 0.00E+00 | 79% |
| [M.Vch77ORF1483P](http://rebase.neb.com/cgi-bin/seqget?M.Vch77ORF1483P) | 694 | 1 | - | 660 | 0.00E+00 | 77% |
| [M.PaeLESBORF23591P](http://rebase.neb.com/cgi-bin/seqget?M.PaeLESBORF23591P) | 707 | 1 | - | 372 | 0.00E+00 | 66% |
| [M.PaeL7ORF11985P](http://rebase.neb.com/cgi-bin/seqget?M.PaeL7ORF11985P) | 707 | 1 | - | 372 | 0.00E+00 | 66% |
| [M.Pae65ORF12290P](http://rebase.neb.com/cgi-bin/seqget?M.Pae65ORF12290P) | 707 | 1 | - | 372 | 0.00E+00 | 66% |
| [M.Pae4ORF12245P](http://rebase.neb.com/cgi-bin/seqget?M.Pae4ORF12245P) | 707 | 1 | - | 372 | 0.00E+00 | 66% |

**Supplementary Table 8**. Top BLASTX hits of M.CmuRB25DamP against the REBASE database.

| **MTase** | **Length** | **Type** | **Specificity** | **Score** | **E-val** | **Identity** |
| --- | --- | --- | --- | --- | --- | --- |
| [M.Sru103234ORFDamP](http://rebase.neb.com/cgi-bin/seqget?M.Sru103234ORFDamP) | 270 | -1 | GATC | 623 | 5.00E-178 | 93% |
| [M.Sma94DamP](http://rebase.neb.com/cgi-bin/seqget?M.Sma94DamP) | 270 | -1 | GATC | 623 | 5.00E-178 | 93% |
| [M.Sli27592DamP](http://rebase.neb.com/cgi-bin/seqget?M.Sli27592DamP) | 270 | -1 | GATC | 620 | 5.00E-177 | 93% |
| [M.Sli21DamP](http://rebase.neb.com/cgi-bin/seqget?M.Sli21DamP) | 270 | -1 | GATC | 620 | 5.00E-177 | 93% |
| [M.SliFK01ORFFP](http://rebase.neb.com/cgi-bin/seqget?M.SliFK01ORFFP) | 270 | -1 | GATC | 618 | 1.00E-176 | 93% |
| [M.SgrA2DamP](http://rebase.neb.com/cgi-bin/seqget?M.SgrA2DamP) | 270 | -1 | GATC | 618 | 1.00E-176 | 93% |
| [M.Spl2CORF7462P](http://rebase.neb.com/cgi-bin/seqget?M.Spl2CORF7462P) | 270 | 2 | GATC | 616 | 5.00E-176 | 92% |
| [M.SspAS9ORF4694P](http://rebase.neb.com/cgi-bin/seqget?M.SspAS9ORF4694P) | 270 | 2 | GATC | 615 | 9.00E-176 | 92% |
| [M.SspAS12ORF4695P](http://rebase.neb.com/cgi-bin/seqget?M.SspAS12ORF4695P) | 270 | 2 | GATC | 615 | 9.00E-176 | 92% |
| [M.SplV4DamP](http://rebase.neb.com/cgi-bin/seqget?M.SplV4DamP) | 270 | 2 | GATC | 615 | 9.00E-176 | 92% |
| [M.SplAS13ORF4695P](http://rebase.neb.com/cgi-bin/seqget?M.SplAS13ORF4695P) | 270 | 2 | GATC | 615 | 9.00E-176 | 92% |
| [M.Spl4RDamP](http://rebase.neb.com/cgi-bin/seqget?M.Spl4RDamP) | 270 | -1 | GATC | 615 | 9.00E-176 | 92% |
| [M.SspTELDamP](http://rebase.neb.com/cgi-bin/seqget?M.SspTELDamP) | 270 | -1 | GATC | 615 | 1.00E-175 | 92% |
| [M.SspSCBDamP](http://rebase.neb.com/cgi-bin/seqget?M.SspSCBDamP) | 270 | -1 | GATC | 615 | 1.00E-175 | 92% |
| [M.SspFS14DamP](http://rebase.neb.com/cgi-bin/seqget?M.SspFS14DamP) | 270 | -1 | GATC | 615 | 1.00E-175 | 92% |
| [M.SmaWW4DamP](http://rebase.neb.com/cgi-bin/seqget?M.SmaWW4DamP) | 270 | -1 | GATC | 615 | 1.00E-175 | 92% |
| [M.SmaSM39DamP](http://rebase.neb.com/cgi-bin/seqget?M.SmaSM39DamP) | 270 | -1 | GATC | 615 | 1.00E-175 | 92% |
| [M.SmaII](http://rebase.neb.com/cgi-bin/seqget?M.SmaII) | 270 | -1 | GATC | 615 | 1.00E-175 | 92% |
| [M.SmaB3R3DamP](http://rebase.neb.com/cgi-bin/seqget?M.SmaB3R3DamP) | 270 | -1 | GATC | 615 | 1.00E-175 | 92% |
| [M.Sma90166DamP](http://rebase.neb.com/cgi-bin/seqget?M.Sma90166DamP) | 270 | -1 | GATC | 615 | 1.00E-175 | 92% |
| [M.Sma836DamP](http://rebase.neb.com/cgi-bin/seqget?M.Sma836DamP) | 270 | -1 | GATC | 615 | 1.00E-175 | 92% |
| [M.Sma66262DamP](http://rebase.neb.com/cgi-bin/seqget?M.Sma66262DamP) | 270 | -1 | GATC | 615 | 1.00E-175 | 92% |
| [M.Sma213ORFBP](http://rebase.neb.com/cgi-bin/seqget?M.Sma213ORFBP) | 270 | -1 | GATC | 615 | 1.00E-175 | 92% |
| [M.Sma14DamP](http://rebase.neb.com/cgi-bin/seqget?M.Sma14DamP) | 270 | -1 | GATC | 615 | 1.00E-175 | 92% |
| [M.Sma14041ORFCP](http://rebase.neb.com/cgi-bin/seqget?M.Sma14041ORFCP) | 270 | -1 | GATC | 615 | 1.00E-175 | 92% |
| [M.Sma107DamP](http://rebase.neb.com/cgi-bin/seqget?M.Sma107DamP) | 270 | -1 | GATC | 615 | 1.00E-175 | 92% |
| [M.Sma1015ORFEP](http://rebase.neb.com/cgi-bin/seqget?M.Sma1015ORFEP) | 270 | -1 | GATC | 615 | 1.00E-175 | 92% |
| [M.SplS13DamP](http://rebase.neb.com/cgi-bin/seqget?M.SplS13DamP) | 270 | 2 | GATC | 613 | 6.00E-175 | 92% |
| [M.SplRVH1ORFAP](http://rebase.neb.com/cgi-bin/seqget?M.SplRVH1ORFAP) | 270 | 2 | GATC | 613 | 6.00E-175 | 92% |

**Supplementary Table 9**. Top BLASTX hits of M.CmuRB25ORF1485P against the REBASE database.

| **MTase** | **Length** | **Type** | **Specificity** | **Score** | **E-val** | **Identity** |
| --- | --- | --- | --- | --- | --- | --- |
| [M.Dsp569ORFEP](http://rebase.neb.com/cgi-bin/seqget?M.Dsp569ORFEP) | 318 | 2 | GATC | 385 | 3.00E-106 | 55% |
| [M.Ddi49ORFBP](http://rebase.neb.com/cgi-bin/seqget?M.Ddi49ORFBP) | 278 | 2 | GATC | 383 | 8.00E-106 | 54% |
| [M1.Ddi453ORFBP](http://rebase.neb.com/cgi-bin/seqget?M1.Ddi453ORFBP) | 278 | 2 | GATC | 383 | 8.00E-106 | 54% |
| [M.DzeEC1ORF12675P](http://rebase.neb.com/cgi-bin/seqget?M.DzeEC1ORF12675P) | 318 | 2 | GATC | 382 | 2.00E-105 | 54% |
| [M.Dze3531ORFCP](http://rebase.neb.com/cgi-bin/seqget?M.Dze3531ORFCP) | 318 | 2 | GATC | 382 | 2.00E-105 | 54% |
| [M.Dze1202ORFBP](http://rebase.neb.com/cgi-bin/seqget?M.Dze1202ORFBP) | 318 | -1 | GATC | 382 | 2.00E-105 | 54% |
| [M.Dze19ORFFP](http://rebase.neb.com/cgi-bin/seqget?M.Dze19ORFFP) | 277 | 2 | GATC | 381 | 3.00E-105 | 54% |
| [M.Dpa2511ORFFP](http://rebase.neb.com/cgi-bin/seqget?M.Dpa2511ORFFP) | 318 | 2 | GATC | 381 | 3.00E-105 | 54% |
| [M.DdaEORF3839P](http://rebase.neb.com/cgi-bin/seqget?M.DdaEORF3839P) | 318 | -1 | GATC | 381 | 3.00E-105 | 54% |
| [M.Dze192ORFDP](http://rebase.neb.com/cgi-bin/seqget?M.Dze192ORFDP) | 318 | 2 | GATC | 380 | 5.00E-105 | 54% |
| [M.Dda898ORFAP](http://rebase.neb.com/cgi-bin/seqget?M.Dda898ORFAP) | 278 | 2 | GATC | 379 | 1.00E-104 | 54% |
| [M.Dsp3274ORFAP](http://rebase.neb.com/cgi-bin/seqget?M.Dsp3274ORFAP) | 318 | 2 | GATC | 378 | 3.00E-104 | 54% |
| [M1.Ddi3534ORFDP](http://rebase.neb.com/cgi-bin/seqget?M1.Ddi3534ORFDP) | 318 | 2 | GATC | 378 | 3.00E-104 | 53% |
| [M.Dda2976ORFCP](http://rebase.neb.com/cgi-bin/seqget?M.Dda2976ORFCP) | 318 | 2 | GATC | 372 | 2.00E-102 | 53% |
| [M1.YpsYPORF2320P](http://rebase.neb.com/cgi-bin/seqget?M1.YpsYPORF2320P) | 272 | 2 | GATC | 363 | 1.00E-99 | 51% |
| [M.Yps7195ORFCP](http://rebase.neb.com/cgi-bin/seqget?M.Yps7195ORFCP) | 272 | 2 | GATC | 360 | 6.00E-99 | 51% |
| [M.Yps447ORF355P](http://rebase.neb.com/cgi-bin/seqget?M.Yps447ORF355P) | 272 | 2 | GATC | 360 | 6.00E-99 | 51% |
| [M.Yps1ORF1410P](http://rebase.neb.com/cgi-bin/seqget?M.Yps1ORF1410P) | 272 | 2 | GATC | 360 | 6.00E-99 | 51% |
| [M2.YpsPBORF1903P](http://rebase.neb.com/cgi-bin/seqget?M2.YpsPBORF1903P) | 272 | 2 | - | 359 | 1.00E-98 | 51% |
| [M.YpsMD67ORF1128P](http://rebase.neb.com/cgi-bin/seqget?M.YpsMD67ORF1128P) | 272 | -1 | GATC | 358 | 2.00E-98 | 51% |
| [M.YpsAORF1863P](http://rebase.neb.com/cgi-bin/seqget?M.YpsAORF1863P) | 272 | -1 | GATC | 358 | 2.00E-98 | 51% |
| [M.YpsMD67ORF59P](http://rebase.neb.com/cgi-bin/seqget?M.YpsMD67ORF59P) | 307 | 2 | GATC | 357 | 8.00E-98 | 51% |
| [M.Yps1ORF335P](http://rebase.neb.com/cgi-bin/seqget?M.Yps1ORF335P) | 307 | 2 | GATC | 357 | 8.00E-98 | 51% |
| [M1.Yal159ORF520P](http://rebase.neb.com/cgi-bin/seqget?M1.Yal159ORF520P) | 272 | 2 | - | 356 | 1.00E-97 | 51% |
| [M.YpsST42ORFEP](http://rebase.neb.com/cgi-bin/seqget?M.YpsST42ORFEP) | 272 | -1 | GATC | 355 | 2.00E-97 | 50% |
| [M.Yps6904ORF444P](http://rebase.neb.com/cgi-bin/seqget?M.Yps6904ORF444P) | 300 | 2 | GATC | 353 | 8.00E-97 | 51% |
| [M.YpsYPORF888P](http://rebase.neb.com/cgi-bin/seqget?M.YpsYPORF888P) | 290 | -1 | GATC | 352 | 3.00E-96 | 48% |
| [M.YruSC09ORF10010P](http://rebase.neb.com/cgi-bin/seqget?M.YruSC09ORF10010P) | 274 | 2 | GATC | 349 | 2.00E-95 | 48% |
| [M.Sma836ORF1018P](http://rebase.neb.com/cgi-bin/seqget?M.Sma836ORF1018P) | 299 | 2 | GATC | 348 | 2.00E-95 | 47% |

**Supplementary Table 10.** Top BLASTX hits of [M.CmuRB25ORF14775P](http://rebase.neb.com/rebase/enz/M.SmuRB25ORF14775P.html) against the REBASE database.

| **MTase** | **Length** | **Type** | **Specificity** | **Score** | **E-val** | **Identity** |
| --- | --- | --- | --- | --- | --- | --- |
| [M.Pae15442ORFBP](http://rebase.neb.com/cgi-bin/seqget?M.Pae15442ORFBP) | 352 | 2 | GTCGAC | 678 | 0.00E+00 | 74% |
| [M.LhuHB2ORFBP](http://rebase.neb.com/cgi-bin/seqget?M.LhuHB2ORFBP) | 352 | 2 | GTCGAC | 678 | 0.00E+00 | 74% |
| [M.Bbr1334ORF2289P](http://rebase.neb.com/cgi-bin/seqget?M.Bbr1334ORF2289P) | 352 | 2 | GTCGAC | 676 | 0.00E+00 | 74% |
| [M.Bth59ORF515P](http://rebase.neb.com/cgi-bin/seqget?M.Bth59ORF515P) | 355 | 2 | GTCGAC | 665 | 0.00E+00 | 73% |
| [M.Bth4ORFHP](http://rebase.neb.com/cgi-bin/seqget?M.Bth4ORFHP) | 355 | 2 | GTCGAC | 665 | 0.00E+00 | 73% |
| [M.BokEORFCP](http://rebase.neb.com/cgi-bin/seqget?M.BokEORFCP) | 355 | 2 | GTCGAC | 661 | 0.00E+00 | 73% |
| [M.BokBDUORF3258P](http://rebase.neb.com/cgi-bin/seqget?M.BokBDUORF3258P) | 355 | 2 | GTCGAC | 661 | 0.00E+00 | 73% |
| [M.XfuRORFBP](http://rebase.neb.com/cgi-bin/seqget?M.XfuRORFBP) | 352 | 2 | GTCGAC | 659 | 0.00E+00 | 72% |
| [M.Xfu4884ORFCP](http://rebase.neb.com/cgi-bin/seqget?M.Xfu4884ORFCP) | 352 | 2 | GTCGAC | 659 | 0.00E+00 | 72% |
| [M.OanML7ORF1065P](http://rebase.neb.com/cgi-bin/seqget?M.OanML7ORF1065P) | 352 | 2 | GTCGAC | 654 | 0.00E+00 | 72% |
| [M.XfasORF2793P](http://rebase.neb.com/cgi-bin/seqget?M.XfasORF2793P) | 387 | 2 | GTCGAC | 636 | 0.00E+00 | 71% |
| [M.XfaOORFC725P](http://rebase.neb.com/cgi-bin/seqget?M.XfaOORFC725P) | 387 | 2 | GTCGAC | 636 | 0.00E+00 | 71% |
| [M.XfaAnn1ORF4130P](http://rebase.neb.com/cgi-bin/seqget?M.XfaAnn1ORF4130P) | 387 | 2 | GTCGAC | 636 | 0.00E+00 | 71% |
| [M.XfaTORF577P](http://rebase.neb.com/cgi-bin/seqget?M.XfaTORF577P) | 355 | 2 | GTCGAC | 635 | 0.00E+00 | 71% |
| [M.XfaM23ORF606P](http://rebase.neb.com/cgi-bin/seqget?M.XfaM23ORF606P) | 355 | 2 | GTCGAC | 635 | 0.00E+00 | 71% |
| [M.Xfa514ORF8380P](http://rebase.neb.com/cgi-bin/seqget?M.Xfa514ORF8380P) | 355 | 2 | GTCGAC | 635 | 0.00E+00 | 71% |
| [M.XfaMDORF2243P](http://rebase.neb.com/cgi-bin/seqget?M.XfaMDORF2243P) | 355 | 2 | GTCGAC | 634 | 0.00E+00 | 71% |
| [M.Xfa34ORF2460P](http://rebase.neb.com/cgi-bin/seqget?M.Xfa34ORF2460P) | 355 | 2 | GTCGAC | 634 | 0.00E+00 | 71% |
| [M.XfaCO33ORF8725P](http://rebase.neb.com/cgi-bin/seqget?M.XfaCO33ORF8725P) | 355 | 2 | GTCGAC | 631 | 3.00E-180 | 70% |
| [M.Avi180ORF1461P](http://rebase.neb.com/cgi-bin/seqget?M.Avi180ORF1461P) | 372 | 2 | GTCGAC | 628 | 2.00E-179 | 69% |
| [M.Psp79ORF4694P](http://rebase.neb.com/cgi-bin/seqget?M.Psp79ORF4694P) | 355 | 2 | GTCGAC | 609 | 1.00E-173 | 66% |
| [M.Psy41aORFAP](http://rebase.neb.com/cgi-bin/seqget?M.Psy41aORFAP) | 355 | 2 | GTCGAC | 606 | 1.00E-172 | 66% |
| [M.Jho1ORF7251P](http://rebase.neb.com/cgi-bin/seqget?M.Jho1ORF7251P) | 375 | 2 | GTCGAC | 568 | 2.00E-161 | 61% |
| [M.Ama292ORFBP](http://rebase.neb.com/cgi-bin/seqget?M.Ama292ORFBP) | 359 | 2 | GTCGAC | 561 | 2.00E-159 | 60% |
| [M.SleC34ORF34350P](http://rebase.neb.com/cgi-bin/seqget?M.SleC34ORF34350P) | 363 | 2 | GTCGAC | 293 | 1.00E-156 | 64% |
| [M.OtrDG6ORF1220P](http://rebase.neb.com/cgi-bin/seqget?M.OtrDG6ORF1220P) | 368 | 2 | GTCGAC | 280 | 2.00E-150 | 60% |
| [M.DspDDS1ORFCP](http://rebase.neb.com/cgi-bin/seqget?M.DspDDS1ORFCP) | 366 | 2 | GTCGAC | 288 | 7.00E-134 | 56% |
| [M.BbrBB02ORFAP](http://rebase.neb.com/cgi-bin/seqget?M.BbrBB02ORFAP) | 355 | 2 | GTCGAC | 283 | 2.00E-132 | 56% |
| [M.NspRr217ORF3797P](http://rebase.neb.com/cgi-bin/seqget?M.NspRr217ORF3797P) | 353 | 2 | GTCGAC | 272 | 6.00E-130 | 55% |

**Supplementary Table 11. Top BLASTX hits of M.CmuRB25ORF23090P against the REBASE database.**

| **MTase** | **Length** | **Type** | **Specificity** | **Score** | **E-val** | **Identity** |
| --- | --- | --- | --- | --- | --- | --- |
| [M.YenYE53ORF411P](http://rebase.neb.com/cgi-bin/seqget?M.YenYE53ORF411P) | 373 | 2 | AAGCTT | 456 | 0.00E+00 | 60% |
| [M.SspM24T3ORF9686P](http://rebase.neb.com/cgi-bin/seqget?M.SspM24T3ORF9686P) | 374 | 2 | AAGCTT | 409 | 8.00E-180 | 56% |
| [M.Pag80401ORF2273P](http://rebase.neb.com/cgi-bin/seqget?M.Pag80401ORF2273P) | 372 | 2 | AAGCTT | 340 | 3.00E-177 | 56% |
| [M.Ecl34399ORF13515P](http://rebase.neb.com/cgi-bin/seqget?M.Ecl34399ORF13515P) | 374 | 2 | AAGCTT | 339 | 4.00E-165 | 53% |
| [M.Sen19443ORF21340P](http://rebase.neb.com/cgi-bin/seqget?M.Sen19443ORF21340P) | 374 | 2 | AAGCTT | 330 | 2.00E-151 | 52% |
| [M.CbrCB01ORF15875P](http://rebase.neb.com/cgi-bin/seqget?M.CbrCB01ORF15875P) | 375 | 2 | AAGCTT | 328 | 2.00E-148 | 52% |
| [M.PsyPaVt10ORFDP](http://rebase.neb.com/cgi-bin/seqget?M.PsyPaVt10ORFDP) | 464 | 2 | AAGCTT | 190 | 2.00E-121 | 42% |
| [M2.PalOT69ORF11160P](http://rebase.neb.com/cgi-bin/seqget?M2.PalOT69ORF11160P) | 451 | 2 | - | 182 | 4.00E-114 | 43% |
| [M.Pae14978ORFGP](http://rebase.neb.com/cgi-bin/seqget?M.Pae14978ORFGP) | 468 | 2 | AAGCTT | 230 | 2.00E-111 | 42% |
| [M.Pae14958ORFEP](http://rebase.neb.com/cgi-bin/seqget?M.Pae14958ORFEP) | 468 | 2 | AAGCTT | 230 | 2.00E-111 | 42% |
| [M.PspHORFCP](http://rebase.neb.com/cgi-bin/seqget?M.PspHORFCP) | 460 | 2 | AAGCTT | 210 | 3.00E-110 | 42% |
| [M.Pae20265ORF1354P](http://rebase.neb.com/cgi-bin/seqget?M.Pae20265ORF1354P) | 460 | 2 | AAGCTT | 210 | 3.00E-110 | 42% |
| [M.PsyB301DORF14465P](http://rebase.neb.com/cgi-bin/seqget?M.PsyB301DORF14465P) | 371 | 2 | AAGCTT | 180 | 6.00E-110 | 39% |
| [M.Psy301ORF5092P](http://rebase.neb.com/cgi-bin/seqget?M.Psy301ORF5092P) | 371 | 2 | AAGCTT | 180 | 6.00E-110 | 39% |
| [M.Xor7342ORFAP](http://rebase.neb.com/cgi-bin/seqget?M.Xor7342ORFAP) | 354 | 2 | AAGCTT | 185 | 9.00E-110 | 40% |
| [M.Csp2512ORFHP](http://rebase.neb.com/cgi-bin/seqget?M.Csp2512ORFHP) | 618 | 2 | - | 128 | 2.00E-108 | 43% |
| [M.AveRAG1ORFEP](http://rebase.neb.com/cgi-bin/seqget?M.AveRAG1ORFEP) | 382 | 2 | AAGCTT | 249 | 7.00E-108 | 38% |
| [M.Pae14978ORFEP](http://rebase.neb.com/cgi-bin/seqget?M.Pae14978ORFEP) | 462 | 2 | AAGCTT | 190 | 7.00E-107 | 41% |
| [M.Pae14958ORFCP](http://rebase.neb.com/cgi-bin/seqget?M.Pae14958ORFCP) | 462 | 2 | AAGCTT | 190 | 7.00E-107 | 41% |
| [M.Pae490ORF11645P](http://rebase.neb.com/cgi-bin/seqget?M.Pae490ORF11645P) | 444 | 2 | AAGCTT | 191 | 7.00E-107 | 41% |
| [M.Bce7H2ORF3355P](http://rebase.neb.com/cgi-bin/seqget?M.Bce7H2ORF3355P) | 425 | 2 | - | 202 | 1.00E-106 | 40% |
| [M.Bce16B4ORF3153P](http://rebase.neb.com/cgi-bin/seqget?M.Bce16B4ORF3153P) | 425 | 2 | - | 202 | 1.00E-106 | 40% |
| [M.SspYBL2ORF13220P](http://rebase.neb.com/cgi-bin/seqget?M.SspYBL2ORF13220P) | 403 | 2 | AAGCTT | 206 | 3.00E-105 | 39% |
| [M.Sya25ORFEP](http://rebase.neb.com/cgi-bin/seqget?M.Sya25ORFEP) | 405 | 2 | AAGCTT | 211 | 1.00E-104 | 40% |
| [M2.OspSJY1ORFFP](http://rebase.neb.com/cgi-bin/seqget?M2.OspSJY1ORFFP) | 431 | 2 | - | 186 | 5.00E-104 | 38% |
| [M.DspCs14ORF2654P](http://rebase.neb.com/cgi-bin/seqget?M.DspCs14ORF2654P) | 464 | 2 | AAGCTT | 195 | 4.00E-102 | 38% |
| [M.BpsSORF1047P](http://rebase.neb.com/cgi-bin/seqget?M.BpsSORF1047P) | 527 | 2 | - | 120 | 1.00E-101 | 40% |
| [M.BpsGSORF4173P](http://rebase.neb.com/cgi-bin/seqget?M.BpsGSORF4173P) | 527 | 2 | - | 120 | 1.00E-101 | 40% |
| [M.BpsAH4ORF3966P](http://rebase.neb.com/cgi-bin/seqget?M.BpsAH4ORF3966P) | 527 | 2 | - | 120 | 1.00E-101 | 40% |

**Supplementary Table 12. Top BLASTx hits of M.CmuRB25ORF22915P against the REBASE database.**

| **MTase** | **Length** | **Type** | **Specificity** | **Score** | **E-val** | **Identity** |
| --- | --- | --- | --- | --- | --- | --- |
| [M.Sen10708ORF7015P](http://rebase.neb.com/cgi-bin/seqget?M.Sen10708ORF7015P) | 278 | 2 | TGGCCA | 384 | 1.00E-138 | 75% |
| [M.Sen633ORF1691P](http://rebase.neb.com/cgi-bin/seqget?M.Sen633ORF1691P) | 280 | 2 | TGGCCA | 383 | 7.00E-137 | 74% |
| [M.Pal30120ORF744P](http://rebase.neb.com/cgi-bin/seqget?M.Pal30120ORF744P) | 265 | 2 | TGGCCA | 486 | 1.00E-136 | 70% |
| [M.PanB19ORFCP](http://rebase.neb.com/cgi-bin/seqget?M.PanB19ORFCP) | 279 | 2 | TGGCCA | 485 | 1.00E-136 | 70% |
| [M.Sen19443ORF5417P](http://rebase.neb.com/cgi-bin/seqget?M.Sen19443ORF5417P) | 280 | 2 | TGGCCA | 382 | 2.00E-136 | 74% |
| [M.Sen236CAORF14640P](http://rebase.neb.com/cgi-bin/seqget?M.Sen236CAORF14640P) | 280 | 2 | TGGCCA | 381 | 3.00E-136 | 74% |
| [M.Sen156CAORF15820P](http://rebase.neb.com/cgi-bin/seqget?M.Sen156CAORF15820P) | 280 | 2 | TGGCCA | 381 | 3.00E-136 | 74% |
| [M.Sen4344ORF17135P](http://rebase.neb.com/cgi-bin/seqget?M.Sen4344ORF17135P) | 276 | 2 | TGGCCA | 382 | 9.00E-136 | 73% |
| [M.PanPA4ORFIP](http://rebase.neb.com/cgi-bin/seqget?M.PanPA4ORFIP) | 279 | 2 | TGGCCA | 480 | 6.00E-135 | 69% |
| [M.KorKO348ORFEP](http://rebase.neb.com/cgi-bin/seqget?M.KorKO348ORFEP) | 280 | 2 | TGGCCA | 374 | 2.00E-133 | 73% |
| [M.Sen15791ORF16305P](http://rebase.neb.com/cgi-bin/seqget?M.Sen15791ORF16305P) | 275 | 2 | TGGCCA | 373 | 8.00E-132 | 71% |
| [M.EicORF3149P](http://rebase.neb.com/cgi-bin/seqget?M.EicORF3149P) | 268 | 2 | TGGCCA | 459 | 1.00E-128 | 67% |
| [M.Eic194ORF15930P](http://rebase.neb.com/cgi-bin/seqget?M.Eic194ORF15930P) | 268 | 2 | TGGCCA | 459 | 1.00E-128 | 67% |
| [M.Eic11ORF15885P](http://rebase.neb.com/cgi-bin/seqget?M.Eic11ORF15885P) | 268 | 2 | TGGCCA | 459 | 1.00E-128 | 67% |
| [M.Yen3502ORF4233P](http://rebase.neb.com/cgi-bin/seqget?M.Yen3502ORF4233P) | 274 | 2 | TGGCCA | 450 | 7.00E-126 | 65% |
| [M2.SspAS9ORF2819P](http://rebase.neb.com/cgi-bin/seqget?M2.SspAS9ORF2819P) | 274 | 2 | TGGCCA | 405 | 2.00E-112 | 57% |
| [M2.SspAS12ORF2820P](http://rebase.neb.com/cgi-bin/seqget?M2.SspAS12ORF2820P) | 274 | 2 | TGGCCA | 405 | 2.00E-112 | 57% |
| [M2.SplAS13ORF2821P](http://rebase.neb.com/cgi-bin/seqget?M2.SplAS13ORF2821P) | 274 | 2 | TGGCCA | 405 | 2.00E-112 | 57% |
| [M.EspUCDORF10265P](http://rebase.neb.com/cgi-bin/seqget?M.EspUCDORF10265P) | 277 | 2 | TGGCCA | 402 | 1.00E-111 | 59% |
| [M.Sen19443ORF21245P](http://rebase.neb.com/cgi-bin/seqget?M.Sen19443ORF21245P) | 287 | 2 | TGGCCA | 398 | 2.00E-110 | 58% |
| [M.CbrCB04ORF18285P](http://rebase.neb.com/cgi-bin/seqget?M.CbrCB04ORF18285P) | 272 | 2 | TGGCCA | 393 | 7.00E-109 | 58% |
| [M.Xor2286ORF21865P](http://rebase.neb.com/cgi-bin/seqget?M.Xor2286ORF21865P) | 287 | 2 | TGGCCA | 319 | 1.00E-108 | 60% |
| [M.PpuS46ORF11480P](http://rebase.neb.com/cgi-bin/seqget?M.PpuS46ORF11480P) | 281 | 2 | TGGCCA | 299 | 1.00E-105 | 56% |
| [M.XalGPEORF178P](http://rebase.neb.com/cgi-bin/seqget?M.XalGPEORF178P) | 282 | 2 | TGGCCA | 312 | 1.00E-104 | 57% |
| [M.Yfr33641ORF4391P](http://rebase.neb.com/cgi-bin/seqget?M.Yfr33641ORF4391P) | 279 | 2 | TGGCCA | 302 | 3.00E-104 | 60% |
| [M.Aba433ORF1524P](http://rebase.neb.com/cgi-bin/seqget?M.Aba433ORF1524P) | 281 | 2 | TGGCCA | 305 | 2.00E-101 | 56% |
| [M.DacSPHORF4704P](http://rebase.neb.com/cgi-bin/seqget?M.DacSPHORF4704P) | 286 | 2 | TGGCCA | 304 | 5.00E-100 | 57% |
| [M.Ppn16536ORF24555P](http://rebase.neb.com/cgi-bin/seqget?M.Ppn16536ORF24555P) | 277 | 2 | TGGCCA | 363 | 6.00E-100 | 53% |
| [M.YpeZORF1740P](http://rebase.neb.com/cgi-bin/seqget?M.YpeZORF1740P) | 232 | 2 | TGGCCA | 362 | 2.00E-99 | 53% |
